# Characteristics and course of patients treated with Kampo Medicine in the Department of General Medicine

**DOI:** 10.1002/jgf2.294

**Published:** 2020-02-21

**Authors:** Shin Takayama, Tetsuya Akaishi, Hiroyuki Nozaki, Satoko Suzuki, Ryutaro Arita, Natsumi Saito, Junichi Tanaka, Takehiro Numata, Akiko Kikuchi, Minoru Ohsawa, Michiaki Abe, Tadashi Ishii

**Affiliations:** ^1^ Department of Kampo Medicine Tohoku University Hospital Sendai Japan; ^2^ Department of Education and Support for Regional Medicine Tohoku University Hospital Sendai Japan; ^3^ Department of Gynecology and Obstetrics Tohoku University School of Medicine Sendai Japan

**Keywords:** characteristics, general medicine, herbal medicine, Japan, Kampo medicine

## Abstract

**Background:**

A recent investigation reported that 92.7% Japanese family physicians have prescribed Kampo medicine (KM). KM can treat a wide variety of conditions from mental disorders to physical weaknesses. However, the characteristics and course of patients treated with KM at the Department of General Medicine remain unclear.

**Aims:**

To investigate the characteristics and course of patients treated with KM in our hospital.

**Methods:**

Data on medical history, complaints, course after Kampo treatment, and Hamilton Depression Rating Scale (HAM‐D) scores were retrogradely collected. The background of patients who received Kampo treatment was compared to that of patients who did not.

**Result:**

Of 362 patients, 51 were treated with KM. Symptoms for which KM was prescribed included pain, general malaise, or sensory disturbance of extremities. All patients treated with KM were screened and initially diagnosed with a functional disorder or noncritical condition. KM including a crude drug of *saiko* such as hochuekkito, shigyakusan, shosaikoto, and yokukansan, was frequently prescribed for patients. Subjective symptoms showed improvement (53%) and no change (47%), while worsening was not observed in any patient. HAM‐D scores showed that patients treated with KM had higher anxiety levels and related symptoms as well as a higher frequency of mental disorders prior to presenting at the hospital.

**Conclusion:**

Most complaints of the patients treated with KM were pain, general malaise, and sensory disturbance. KM is more likely to be prescribed in patients with health‐related anxiety or a history of mental disorders.

## Graphical abstract

Kampo medicine is more likely to be prescribed in patients with health‐related anxiety or a history of mental disorders who visit the Department of General Medicine.

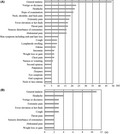

## 1 INTRODUCTION

Kampo is a traditional Japanese medicine that was imported from China between the fifth and sixth centuries.^1^ Currently, physicians are permitted to use basic Kampo prescriptions that have been covered by national Japanese government health insurance in Japan. In total, 148 Kampo prescriptions may be provided under medical license according to the Ministry of Health, Labour and Welfare of Japan.^2^ A recent investigation reported that 92.7% Japanese family physicians have started to prescribe Kampo medicine (KM).^3^ Considering the growing evidence on KM,^4^ several clinical practice guidelines (CPG) have listed KM as a treatment modality to address many conditions, disorders, and diseases,^5^, ^6^, ^7^, ^8^ such as dementia, behavioral and psychological symptoms of dementia, prevention of aspiration pneumonia, gut disorders, and menstrual symptoms. Furthermore, several articles have reported the economic advantages of administering a Kampo prescription, including reduced hospitalizations and medical costs.^9^, ^10^, ^11^, ^12^, ^13^, ^14^ The Japanese Ministry of Education, Culture, Science, Sports and Technology revised the model core curriculum for Japanese medical education to include KM in 2001,^15^ which has subsequently been incorporated into the medical education curriculum in 80 Japanese medical schools and universities in 2007.^16^ The World Federation for Medical Education Global Standards for Quality Improvement, which evaluates the education of complementary and alternative medicine or traditional medicine in each university in Japan.^17^ has also admitted the need of KM. The World Health Organization published International Statistical Classification of Diseases and Related Health Problems, 11th Revision, which included Supplementary Chapter Traditional Medicine Conditions‐Module I in 2018.^18^ It is possible that a physician may prescribe KM considering the various factors associated with the same, such as available clinical evidence, CPG, cost‐effective technique without the need for hospitalization, and acceptance by both the medical education curriculum and medical education evaluation standards along with the current global trends.

We have previously reported that patients that visited the Department of General Medicine have had numerous issues.^19^ Multiple Western medicine prescriptions were typically needed to treat multiple symptoms; however, recent CPG have suggested the risk of polypharmacy considering its adverse effects.^6^ At the first visit, patients with unidentified and multiple complaints, including general malaise who present at general hospitals, are usually assessed and treated in our department. In cases with patients registering several complaints, physicians typically prescribe Ktiple phyM since it includes mulsiological substances and can improve the primary complaint along with the accompanying symptoms. Although patients are eventually treated with KM, until now, the demographic and psychological backgrounds and characteristics of patients treated with KM and the course after the treatment have not been evaluated. Thus, in this study, we aimed to evaluate the characteristics and the course after the treatment in patients treated with KM.

## 2 MATERIALS AND METHODS

### 2.1 Study design

Retrospective observational study.

### 2.2 Data collection

#### 2.2.1 Inclusion criteria

All patients who visited the Department of General Medicine in the Tohoku University Hospital between April 2016 and May 2017 were enrolled in this study. Comprehensive data were collected on each patient, including their chief complaints, medical history, history of treatment with KM, initial diagnosis, type of prescribed KM, course after Kampo treatment, other relevant clinical information, and the Hamilton Depression Rating Scale (HAM‐D) that evaluates depressive mood, anxiety, and psychosomatic symptoms.

#### 2.2.2 Exclusion criteria

Hamilton Depression Rating Scale was performed for all patients who visited our department to confirm the presence of depressive mood, anxiety, and psychosomatic conditions. However, the patients who suffered from emergency conditions or those who declined HAM‐D could not be evaluated via HAM‐D. We could not use the HAM‐D data of these patients; therefore, we excluded patients without HAM‐D from the subanalysis.

### 2.3 Clinical setting

Our department is comprised of ten doctors. There are seven internists, one surgeon, one gynecologist, and one general practitioner. Of the ten doctors, two internists and the gynecologist specialized in KM. The treatment modalities designed by Kampo‐specialized doctors depended on specialized Kampo theory, which included the patterns of yan‐ying, qi‐blood‐fluid, heat‐cold, kyo‐jitsu, six‐stage, and evidence‐based medicine (EBM) along with CPG associated with KM. However, considering the fact that the other doctors had a little knowledge of specialized Kampo theory to an extent, these doctors selected Kampo prescription according to KM‐associated EBM and CPG. Characteristically, our department has several multisectional doctors with a varied degree of clinical experience, specialty, or knowledge associated with KM. This is one of the primary reasons why it is not possible to unify the differences and the variations involved in identifying Kampo prescriptions. Therefore, KM was prescribed based on the doctor's experience, evidence, CPG, medical background of the patient, the presence or absence of Kampo theory, and patients' attitude toward the treatment.

Three levels of subjective symptom evaluation were performed: improvement, no change, and worsened symptoms, to determine the course after treatment when the patient revisited. HAM‐D was evaluated by H. N., a clinical psychologist, and the nurses in the hospital.

### 2.4 Statistical analysis

The patients were categorized into two groups based on the presence or absence of treatment with KM. The Kampo group included the patients who were treated with KM after the initial diagnosis. The patients in this group were defined after being prescribed KM to address their respective symptoms for a minimum of 7 days; furthermore, a combination of Western and KM was permitted. Patients in the non‐Kampo group included those who were not treated with KM.

Demographic and psychological background data were collected and compared between the two groups. Scores from the HAM‐D subscale were compared using the Mann‐Whitney *U* test. Other demographic data were compared using either a Student's *t* test or chi‐square test. In each comparison, a *P*‐value of <.05 was considered to be statistically significant. Statistical analyses in this study were conducted using SPSS Statistics Base 22 software (IBM), JMP Pro 14 (SAS Institute Inc, Cary, NC, USA), and MATLAB R2015a (MathWorks).

### 2.5 Ethical considerations

This study was approved by the Institutional Review Board of the Tohoku University Graduate School of Medicine (Approval Number: 2016‐1‐416). The study protocol was open to the public on the website of Tohoku University (http://www.med.tohoku.ac.jp/public/ekigaku.html) during the research period, and opt‐out method was applied to obtain informed consents.

## 3 RESULTS

### 3.1 Comparison of patient background characteristics

**Figure 1:**
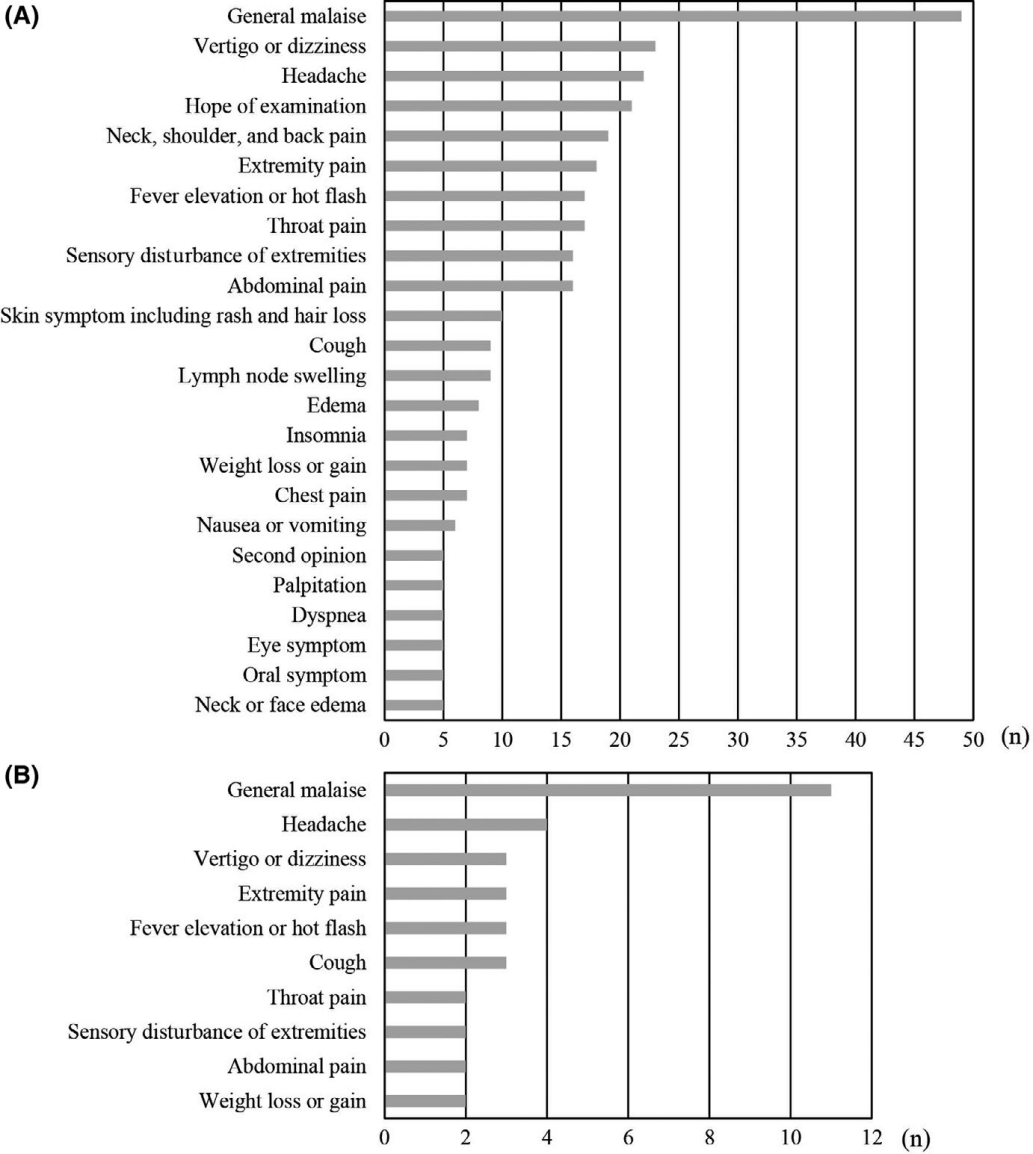
Subjective symptoms of patients visiting the Department of General Medicine. Symptoms presenting in more than five patients are listed in A. Subjective symptoms of patients treated with Kampo medicine. Symptoms presenting in more than 2 patients are listed in B

In total, 362 patients (Average age of 52.8 with SD of 20.2 years) were enrolled in this study, of which 81% received an initial diagnosis. Some patients were introduced to a specific department for treatment options, while others who had critical diseases were ruled out. Patients who visited our department had subjective symptoms (Figure 1A), of which some symptoms were treated with KM (Figure 1B).

**Figure 2:**
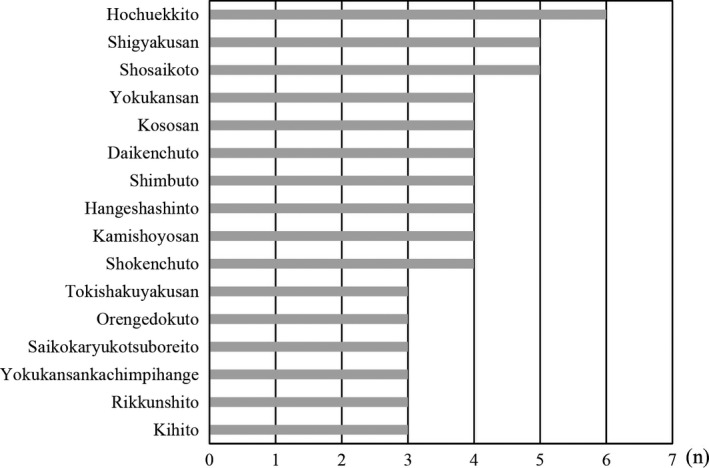
Prescription of Kampo medicine in the Department of General Medicine. Kampo medicines prescribed for more than 3 cases are listed

Of these participants, 51 patients (average age of 51.8 with SD of 21.0 years) were treated with KM (Kampo group) and the other 311 participants were not (non‐Kampo group). We analyzed the medical record of patients in the non‐Kampo group and confirmed that they used neither prescription nor over‐the‐counter KM. Of the 51 patients, 69% were diagnosed with a functional disorder. The others were diagnosed with uncertain but noncritical conditions. Most of the patient complaints treated with KM included pain (49%), general malaise (22%), and sensory disturbance (8%) (Figure 1B). All patients treated with KM were screened and initially diagnosed with a functional or a noncritical condition. KM includes a crude drug of *saiko* (root of *Bupleurum falcatum*) and comes in forms known as Bupleurum Root drugs: hochuekkito, shigyakusan, shosaikoto, and yokukansan. These drugs were frequently prescribed for the patients (Figure 2). Overall, subjective symptom showed improvement (53%), no change (47%), and worsening (0%). 14% of the patients with improvement in symptoms were introduced to the Department of KM, a specialized department that deals with prescribing KM. They all hoped for continuous Kampo treatment.

Demographic data and mean HAM‐D scores for each group are presented in Table 1. Both the HAM‐D mean score and the frequency of preexisting mental disorders were found to be slightly higher in the KM group, but the frequency of using psychiatric drugs was not significantly different between the groups.

**Table 1:** Patient background characteristics by later history of Kampo medicine treatment

Visited the Department of General Medicine		
n	362	
Male:female	194:216	
Age	54.1 ± 19.6	

	Kampo group	Non‐Kampo group	*P*‐value
n	51	311	—
Male: female	23:28	174:188	.77
Age	54.6 ± 19.4	53.8 ± 19.8	.82
Answered all HAM‐D items			
n	32	194	—
Male:female	16:16	86:108	.55
HAM‐D total^a^	10 [6‐17]	8 [4‐15]	.084
History of mental disorders	13 (40.6%)	44 (22.7%)	.030
Use of psychiatric drugs at consultation^b^	2 (6.3%)	9 (4.6%)	.66
Treated by a psychiatrist at consultation	1 (3.1%)	5 (2.6%)	1.00
Eventual follow‐up period [months]	8.5 ± 2.8	8.0 ± 3.2	.47

Abbreviation: HAM‐D, Hamilton Depression Rating Scale.

a Distribution of the HAM‐D total score is represented by the mean and interquartile range [25th–75th percentiles].

b Including sleeping drugs. Age and follow‐up period are shown with the mean ± SD.

### 3.2 Comparisons of the HAM‐D subscale score

**Figure 3:**
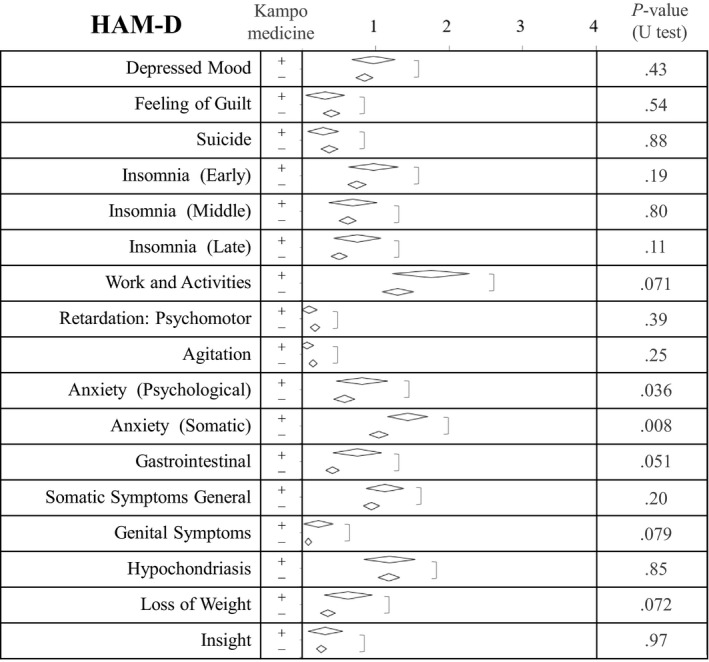
Comparisons of Hamilton Depression Rating Scale (HAM‐D) subscale score by participant history of receiving Kampo medicine treatment. Anxiety with somatic and psychological symptoms was stronger for those who later received Kampo medicine treatments. The *P*‐values were calculated using the Mann‐Whitney *U* test

Of the 362 participants, 226 responded to all HAM‐D questionnaire items. Of the total responders, 32 were a part of the Kampo group, and 194 were a part of the non‐Kampo group. The average and the 95% confidence interval score for each HAM‐D subscale score are listed in Figure 3. Analysis indicated that anxiety (with both somatic and psychological symptoms) before initiating medical treatment was significantly higher in the Kampo group. There were no other significant between‐group differences in the HAM‐D subscale score.

## 4 DISCUSSION

This study revealed that KM was used for functional disorders or uncertain but noncritical conditions following a screening examination at our department. Most of complaints of the patients treated with KM were pain, general malaise, and sensory disturbance. Bupleurum Root drugs were the most frequently prescribed. Over a half of patients treated with Kampo showed improvement in subjective symptoms. KMs are likely to be prescribed to patients who have higher levels of health‐related anxiety or a previous history of mental disorders.

The present study is the first to show the characteristics and course of the patients treated by KM in a Department of General Medicine in a hospital. Based on our findings, we speculate that patients who visited this department may show high levels of anxiety because of unidentified diagnosis following frequent examination in other clinics or hospitals.^19^ After excluding serious or emergent diseases following a comprehensive medical examination, some patients still have complaints about functional but undefined symptoms. In these cases, KM can be prescribed as a treatment option. Bupleurum Root drugs have been prescribed for general malaise, appetite loss, and fever. A randomized controlled trial showed that hochuekkito, including *saiko*, can improve chronic inflammation, general malaise, appetite loss, and malnutrition.^20^, ^21^, ^22^ We also reported that shosaikoto, including *saiko*, improves myalgic encephalomyelitis/chronic fatigue syndrome with chronic febricula.^20^, ^21^, ^23^ Medical insurance adaptation of hochuekkito, shigyakusan, shosaikoto, and yokukansan is listed in Table 2.^20^, ^21^, ^24^ Characteristics of KM that is permitted for use as part of the Japanese health insurance system include multiple herbs that are easy to use and administer (ie, as extracted glandules or pills). These medicines can be used to treat functional symptoms along with mental disorders. For instance, shigyakusan can improve abdominal pain and associated symptoms (eg, feeling full, irritation, and a cold sensation in the extremities). In a case that could have been treated with shigyakusan, the patient complained of abdominal pain and a simultaneous irritation and a cold sensation in the extremities and polypharmacy was required to address multiple symptoms in Western medicine. However, gastrointestinal motility, mood disturbance, and circulation and feeling in extremities can be treated using a single Kampo prescription, with a given condition that these symptoms should not be etiologically associated with organic diseases. In the absence of an organic disease, KM can successfully balance qi‐blood‐fluid and/or heat‐cold condition, which is conceptually different from Western medical practices. Arai et al reported that Kampo prescription to manage psychological symptoms such as yokukansan, kamishoyosan, and kososan was used for psychopathological patients suffering from chronic pain in a multidisciplinary pain center.^25^ For example, yokukansan can influence several neurotransmitters and receptors such as serotonergic^26^, ^27^, ^28^, ^29^, ^30^, ^31^ and glutamate^32^, ^33^, ^34^, ^35^, ^36^, ^37^, ^38^ receptors, which can provide a significant degree of control over the entire body. KM can contribute to controlling anxiety with somatic and psychological symptoms, even when the patient's condition remains unidentified.

**Table 2:** Medical insurance adaptation of Kampo medicine including a crude drug of *saiko*: hochuekkito, shigyakusan, shosaikoto, and yokukansan

	JUNKOU	OHSUGI	TEIKOKU	SANWA	TSUMURA
Hochuekkito	Symptoms of patients who are dispirited, having poor gastrointestinal function, and who are likely to get tired: infirm constitution, fatigue and malaise, declined constitution after disease, anorexia, and night sweats			Symptoms of patients who have a delicate constitution, anemia, reduced gastrointestinal functions, fatigue and malaise, anorexia, perspiration during sleep, etc: debility after disease or surgery, reinforcement of physical strength after chest disease, anemia, hypotension, summer emaciation, dyspepsia, weak gastrointestinal functions, and hyperhidrosis	Indicated for the following symptoms/conditions of patients having delicate constitution, reduced digestive functions, and severe fatigability of limbs: summer emaciation, reinforcement of physical strength after illness, tuberculosis, anorexia, gastroptosis, cold, hemorrhoid, and anal prolapse.
Shigyakusan					Indicated for the relief of the following symptoms of those patients with a comparatively strong constitution: cholecystitis, cholelithiasis, gastritis, hyperacidity, gastric ulcer, nasal catarrh, bronchitis, nervousness, and hysteria.
Shosaikoto	1. Shosaikoto is indicated for the relief of the following symptoms of those patients with moderately strong constitution, right upper abdominal tenderness accompanied by fullness and discomfort, coated tongue, oral cavity discomfort, and anorexia, and/or those with slight fever and nausea. Also indicated for various acute febrile diseases, pneumonia, bronchitis, common cold, lymphadenitis, chronic gastrointestinal disorder, and insufficient postpartum recovery 2. Shosaikoto is indicated for the improvement of liver dysfunction due to chronic hepatitis.				
Yokukansan		Symptoms of patients with delicate constitution and nervousness: neurosis, insomnia, night cry in children, and peevishness in children			TJ‐54 is indicated for the relief of the following symptoms of those patients with delicate constitution and nervousness: neurosis, insomnia, night cry in children, and peevishness in children

Note

JUNKOU, OHSUGI, TEIKOKU, SANWA, and TSUMURA are pharmaceutical companies producing Kampo medicine in Japan [20].

This study has several limitations. First, although it revealed the characteristics of patients who were prescribed KM, it did not evaluate the effectiveness of the use of KM as a whole. Consequently, future research should evaluate the effects of prescribed KM on each condition and symptom. Second, the degree of the Kampo diagnostic and treatment techniques varied significantly considering the fact that our department housed several multisectional specialist doctors. Our department had weekly meetings to present cases of all patients that visited the department for the first time. Additionally, to ensure that the Kampo treatment strategy succeeds, Kampo‐specialized doctors confirmed and suggested strategies of prescribing KM for each patient. Third, in our department, the patients requiring treatments for psychological disorders were introduced to the department of psychiatry in our hospital or clinic. However, we cannot deny the fact that physicians in our department preferred to provide a Kampo prescription to patients who do not seem to need treatments for psychological disorders. Since KM sometimes can sparingly improve psychopathological conditions, it is impossible to ignore the selection bias associated with KM. Fourth, physicians evaluated the treatment outcomes after 2 weeks from the administration of Kampo prescription. However, a number of patients visited from 1 to 4 weeks according to their convenience. Physicians evaluated treatment outcomes on the following visit. We could not control their visiting time according to the patients' convenience. Heterogenic assessments of treatment outcome were taken into account for assessment bias.

## 5 CONCLUSION

Kampo medicine was used for functional disorders or uncertain but noncritical conditions after a screening examination at the Department of General Medicine of Tohoku University Hospital. Most complaints of the patients treated with KM were pain, general malaise, and sensory disturbance. KMs are likely to be prescribed to patients with anxiety or a history of mental disorders. The effectiveness of prescribed KMs for treating these conditions remains to be evaluated.

## CONFLICTS OF INTEREST

Takayama S., Kikuchi A., Ohsawa M., and Ishii T are members of the Department of Kampo Medicine, Tohoku University Graduate School of Medicine, which has a joint research collaboration with Tsumura & Co. (Tokyo, Japan).The other authors have stated explicitly that there are no conflicts of interest in connection with this article.

## AUTHOR CONTRIBUTIONS

Takayama S. designed this study and wrote the manuscript. Akaishi T analyzed the data and wrote the manuscript. Nozaki H examined and evaluated HAM‐D and collected patient data. Suzuki S., Arita R., Saito N., Tanaka J., Abe M., and Ishii T. provided their valuable advice for drafting the manuscript. Numata T., Kikuchi A., and Ohsawa M. supported the Kampo treatment in the clinical setting.
